# Linked color imaging improves identification of early gastric cancer lesions by expert and non-expert endoscopists

**DOI:** 10.1007/s00464-022-09280-0

**Published:** 2022-05-04

**Authors:** Kiki Fockens, Jeroen de Groof, Joost van der Putten, Tsevelnorov Khurelbaatar, Hisashi Fukuda, Takahito Takezawa, Yoshimasa Miura, Hiroyuki Osawa, Hironori Yamamoto, Jacques Bergman

**Affiliations:** 1 Department of Gastroenterology and Hepatology, Amsterdam UMC, University of Amsterdam, Amsterdam, The Netherlands; 2 Amsterdam Gastroenterology Endocrinology Metabolism, Amsterdam, The Netherlands; 3 Department of Electrical Engineering, Eindhoven University of Technology, Eindhoven, the Netherlands; 4 Division of Gastroenterology, Department of Medicine, Jichi Medical University, Shimotsuke, Tochigi Japan

**Keywords:** Early gastric cancer, Linked color imaging, Optical enhancement

## Abstract

**Background and aims:**

Early gastric cancer (EGC) lesions are often subtle and endoscopically poorly visible. The aim of this study is to evaluate the additive effect of linked color imaging (LCI) next to white-light endoscopy (WLE) for identification of EGC, when assessed by expert and non-expert endoscopists.

**Methods:**

Forty EGC cases were visualized in corresponding WLE and LCI images. Endoscopists evaluated the cases in 3 assessment phases: Phase 1: WLE images only; Phase 2: LCI images only; Phase 3: WLE and LCI images side-to-side. First, 3 expert endoscopists delineated all cases. A high level of agreement between the expert delineations corresponded with a high AND/OR ratio. Subsequently, 62 non-experts indicated their preferred biopsy location. Outcomes of the study are as follows: (1) difference in expert AND/OR ratio; (2) accuracy of biopsy placement by non-expert endoscopists; and (3) preference of imaging modality by non-expert endoscopists.

**Results:**

Quantitative agreement between experts increased significantly when LCI was available (0.58 vs. 0.46, *p* = 0.007). This increase was more apparent for the more challenging cases (0.21 vs. 0.47, *p* < 0.001). Non-experts placed the biopsy mark more accurately with LCI (82.3% vs. 87.2%, *p* < 0.001). Again this increase was more profound for the more challenging cases (70.4% vs. 83.4%, *p* < 0.001). Non-experts indicated to prefer LCI over WLE.

**Conclusion:**

The addition of LCI next to WLE improves visualization of EGC. Experts reach higher consensus on discrimination between neoplasia and inflammation when using LCI. Non-experts improve their targeted biopsy placement with the use of LCI. LCI therefore appears to be a useful tool for identification of EGC.

**Graphical abstract:**

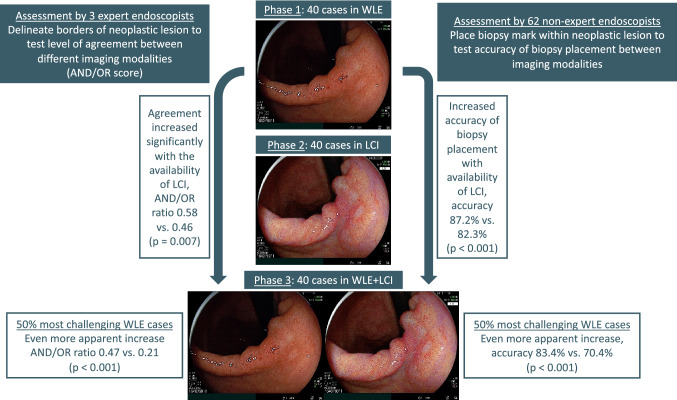

Survival rates of patients with gastric cancer remain poor. When detected at an early stage, patients can be treated endoscopically with good outcomes. However, detection of early gastric cancer (EGC), especially flat lesions, can be difficult with conventional white-light endoscopy (WLE) alone [1, 2]. Important endoscopic characteristics of EGC include subtle change in color of the mucosa and mild elevation and shallow depression.

In the last decade several optical chromoscopy techniques have been developed for detection of early gastrointestinal neoplasia, such as narrow band imaging (NBI, Olympus, Tokyo, Japan), blue-light imaging (BLI, Fujifilm, Tokyo, Japan), and optical enhancement (OE, Pentax Medical, Tokyo, Japan). These techniques generally use short-wavelength excitation light which penetrates more superficially into the tissue with less scattering. The blue/violet excitation light is also highly absorbed by hemoglobin. These features allow better visualization of mucosal and vascular patterns. Studies suggest that these techniques mainly serve as characterization tools for detailed inspection after primary detection of lesions with WLE or for guiding endoscopic resection [3–5].

Linked color imaging (LCI, Fujifilm, Tokyo, Japan) is a recently developed imaging technique which enhances differences in red-to-white color spectrum of images [6]. LCI may improve the primary detection of early gastric cancer, given that these lesions often occur against a background of atrophic gastritis and their distinction relies on subtle differences in the red-to-white spectrum [7–9]. Early case series support this hypothesis [10, 11] and a recent cohort study from 2020 by Yamaoka et al. showed an improvement in detection rate of early gastric cancer lesion and gastric adenomas when using LCI [12].

The aim of this study is to evaluate the additive effect of LCI next to WLE for the identification of early gastric cancer lesions in overview, when assessed by both expert and non-expert endoscopists.

## Methods

### Setting and design

This study was a collaboration between the departments of Gastroenterology and Hepatology of Amsterdam UMC, location AMC, Amsterdam, the Netherlands and Jichi Medical University, Tochigi, Japan. Both centers are specialized in imaging, diagnosis, and treatment of early gastrointestinal neoplasia. The Medical Research Involving Human Subjects Act did not apply to this study. Official approval was therefore waived by the Medical Ethics Review Committee.

### ELUXEO 7000 endoscopy system

All cases for this study were recorded with the ELUXEO 7000 endoscopy system (Fujifilm, Tokyo, Japan). This new-generation endoscopy system facilitates optical chromoscopy, using a 4 light-emitting diode (LED) light source, each LED containing different wavelengths. The imaging modalities of WLE, BLI, and LCI are created by altering the intensity of the LEDs. LCI has been developed to differentiate red color tones more effectively, using a combination of both pre-processing and post-processing techniques with a peak wavelength of 410 nm.

### Acquisition of endoscopic images

**Fig. 1:**
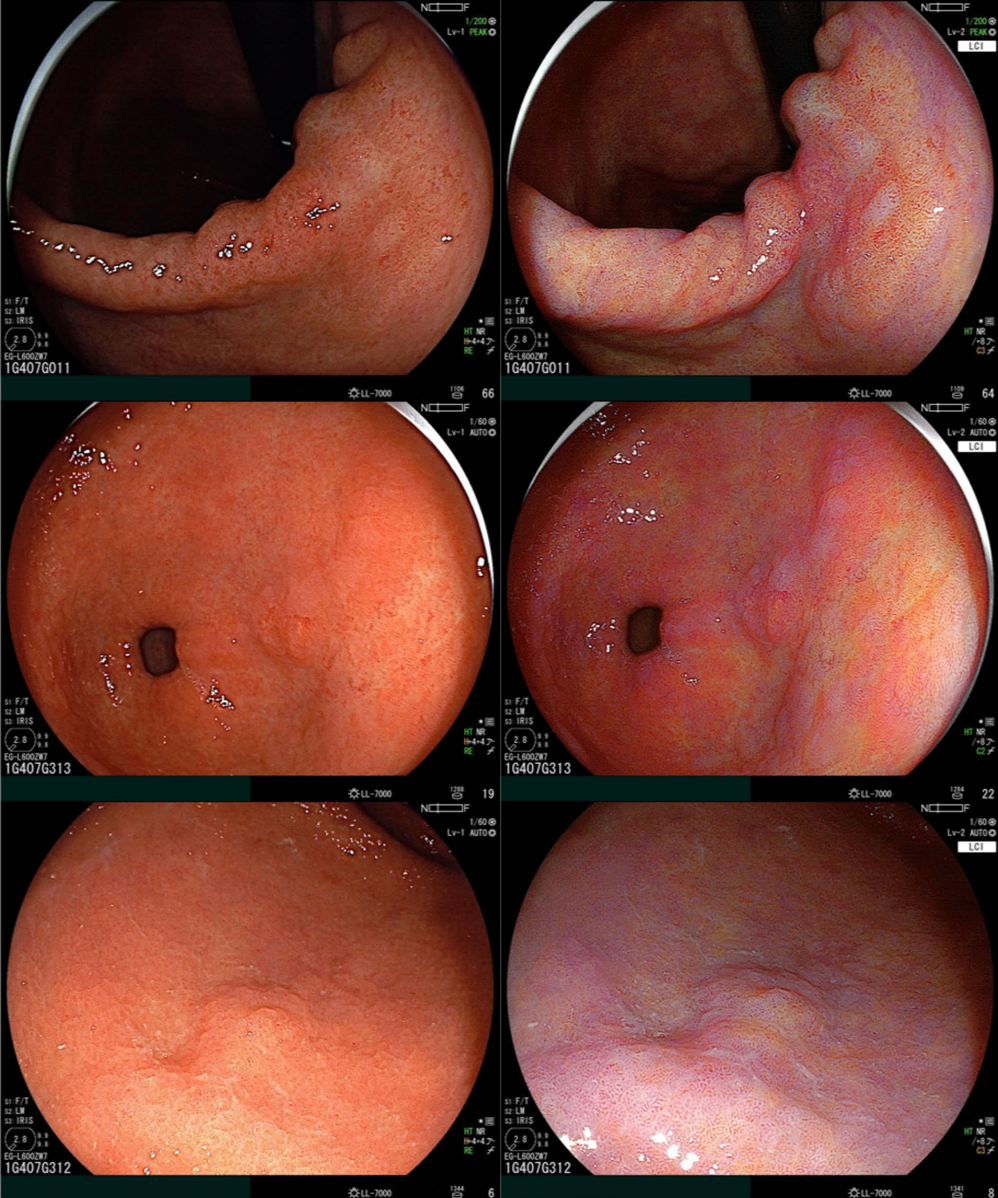
Exemplary cases of early gastric cancer lesions visualized in overview, by corresponding images in white-light endoscopy (left) and linked color imaging (right)

Endoscopic images of early gastric cancer lesions were retrospectively collected from a database at Jichi Medical Center, Japan. This database contains information and images of all patients who underwent endoscopic submucosal dissection for early gastric cancer between June 2018 and January 2019. All cases were histopathological confirmed to contain early gastric neoplasia. For this study, we selected cases with corresponding WLE and LCI images, collected between December 2018 and March 2019. Cases were only included if images of both modalities were obtained with the endoscope in the same position at similar angles. All images were fully anonymized and saved in full HD mode in a web-based module (1280 × 1024 pixels) (Fig. 1).

### Web-based delineation module and outline of assessment phases

An online module (Meducati AB, Göteborg, Sweden) was developed specifically for this study, using software previously described by our group [13, 14]. The module allowed expert and non-expert endoscopists in the field of early gastric cancer to evaluate and score endoscopic images and delineate neoplastic lesions on these images in three separate assessment phases. In the first assessment phase, 50% of the cases were assessed in WLE and the other 50% were presented in LCI. The second assessment phase was set up similar to the first phase; however, the cases assessed in WLE in the first phase were now displayed in LCI and the cases assessed in LCI in the first phase were now displayed in WLE. In the third and final assessment phases, all cases were presented in WLE and LCI in a side-to-side display.

The three assessment phases were separated by a wash-out period of at least two weeks to enable evaluation of the imaging technique and to eliminate a possible learning curve by endoscopists. The order of images was randomized between assessors and assessment phases. Images were locked directly after assessment, and assessors were therefore not able to go back to previous images. Each assessment phase had to be completed in a single session.

For final statistical analyses, all included images and their assessments were rearranged into three classes: 1) WLE; 2) LCI; and 3) combined assessment of WLE and LCI. Using this rearrangement, it was possible to directly compare imaging modalities.

### Assessment by expert endoscopists

Between June 2019 and November 2021, three Japanese expert endoscopists (HF, TT, and YM) in the field of gastric cancer completed all three assessment phases. The participating expert endoscopists had over 10 years of endoscopic experience with gastric cancer, over 2 years of experience with LCI, and all perform endoscopic treatment of gastric cancer on a daily basis. The expert endoscopists were asked to delineate the neoplastic lesion on all images. The surface area of the delineation was expressed as the absolute number of pixels within the delineation.

**Fig. 2:**
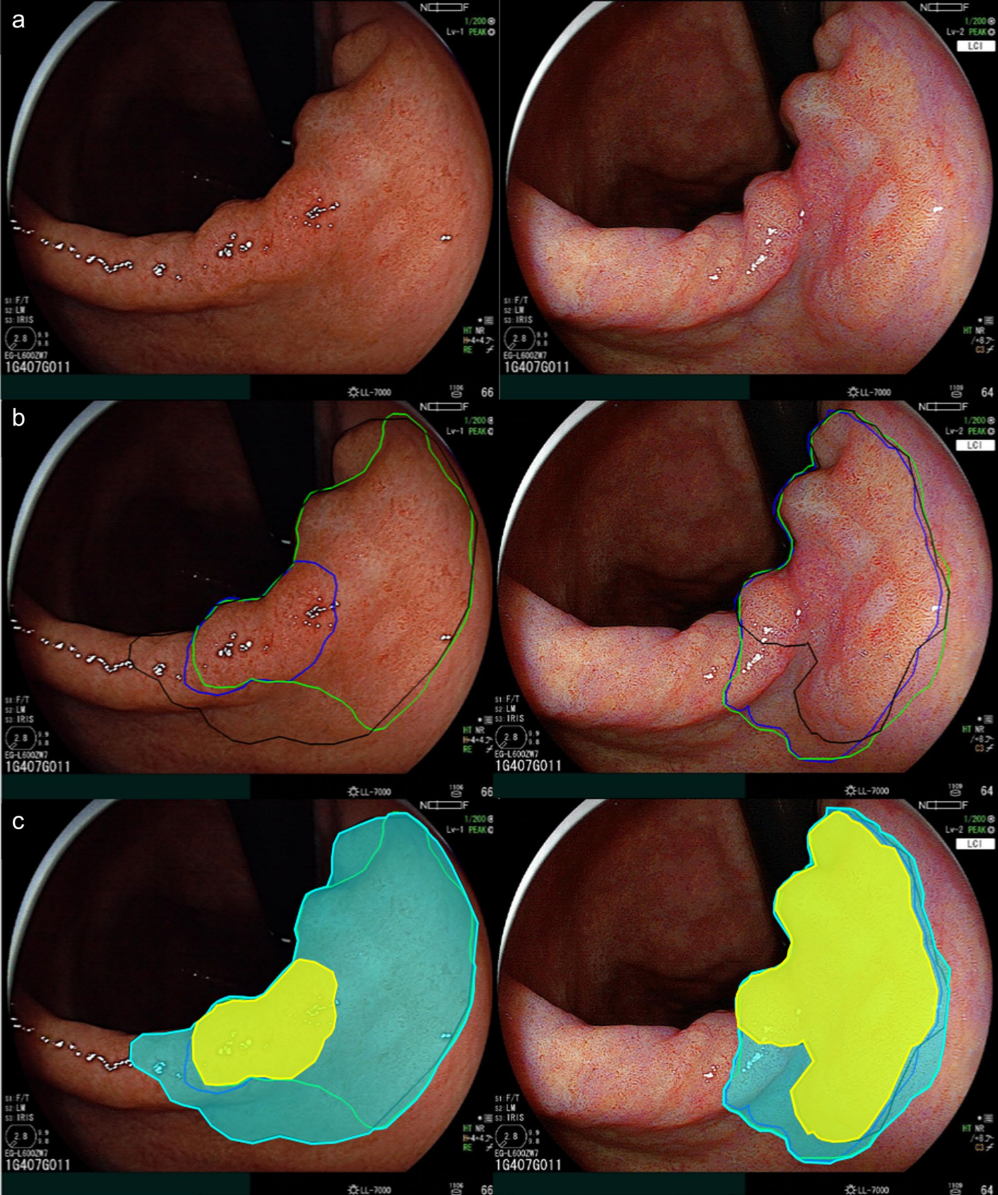
Exemplary case of early gastric cancer lesion **A** visualized in corresponding images in white-light endoscopy (left) and linked color imaging. **B** Delineations of 3 experts displayed in green, blue, and black. **C** AND area visualized in yellow, OR area visualized in blue

The rationale to include expert delineations in this study was twofold. First, this enabled ground truth comparison with non-expert assessors. Furthermore, it enabled direct comparison of expert agreement on their respective delineations when using WLE and LCI. To quantify the agreement between expert delineations, both the “AND area” and the “OR area” were calculated. The “AND area” was defined as the area where the three expert delineations overlapped. This area was considered to contain the most relevant part of the neoplastic lesion. The area delineated by at least one of the experts was labeled as the “OR area” (Fig. 2). The AND/OR ratio was used to reflect the level of agreement between the experts for their delineations. A high AND/OR ratio corresponds with a high level of agreement between expert delineations.

### Assessment by non-expert endoscopists

From June 2019 to December 2019, a group of international endoscopists completed the assessment module. The endoscopists included in the non-expert group were general endoscopists without a specific expertise in gastric cancer. The non-expert endoscopists were divided over three different levels based on their endoscopic expertise: 1. Fellow: gastroenterologists in training; 2. Junior: gastroenterologists with less than 3 years of experience; and 3. Senior: gastroenterologists with over 5 years of experience. The assessors originated from Japan, the Netherlands, Portugal, and Sweden.

In all three assessment phases, the non-expert endoscopists were asked to place a targeted biopsy mark on the most abnormal part of the lesion within the image. This place was supposed to represent the location of the mucosa where they would have taken a targeted biopsy during real-time endoscopic examination. Biopsy position was considered correct when placed within the expert AND area.

In each assessment phase, the non-expert endoscopists were asked to score their ability to delineate the neoplastic lesion in the given imaging modality using a VAS score, ranging from 0 (very hard to delineate) to 10 (very easy to delineate). In assessment phase 3, the non-expert endoscopists were also asked to indicate which imaging modality allowed them best to delineate the neoplastic lesions. An ordinal scale was used, ranging from -2 (LCI is much worse than WLE), -1 (LCI is a little worse than WLE), 0 (LCI is the same as WLE), + 1 (LCI is a little better than WLE), to + 2 (LCI is much better than WLE).

### Outcome measures

#### Expert delineation performance

- Difference in experts AND/OR ratio when using WLE, LCI, or WLE + LCI.

#### Non-expert performance

- Accuracy of the targeted biopsy placement when using WLE, LCI, or WLE + LCI;
- Ability to delineate the neoplastic lesion when using WLE, LCI, or WLE + LCI (VAS scores; ranging 1–10);
- Preferred imaging modality (ordinal scores; ranging from -2 to + 2): WLE vs. LCI comparison.

### Statistical analyses

Statistical analysis was performed using SPSS Statistical software package for Windows (version 25, SPSS Inc., Chicago). For descriptive statistics, normally distributed data were shown as mean (± standard deviation) and variables with skewed distribution were shown as median (interquartile range [IQR]). To compare the difference in AND/OR ratio and to evaluate the ability to delineate the neoplastic lesion per imaging modality, paired analyses were performed using a Wilcoxon-Signed Rank test. To analyze change in targeted biopsy placement, the McNemar test was used for paired data.

## Results

Forty cases of early gastric cancer of 40 unique patients were included in this study. These cases were selected out of a total of 57 patients who underwent an endoscopic submucosal dissection for early gastric cancer between June 2018 and January 2019. Seventeen cases were excluded because of low image quality or lack of similarity between the WLE and LCI image. All lesions were resected after image acquisition and histopathology showed all resection specimens to contain early gastric cancer.

### Expert delineation performance

**Fig. 3:**
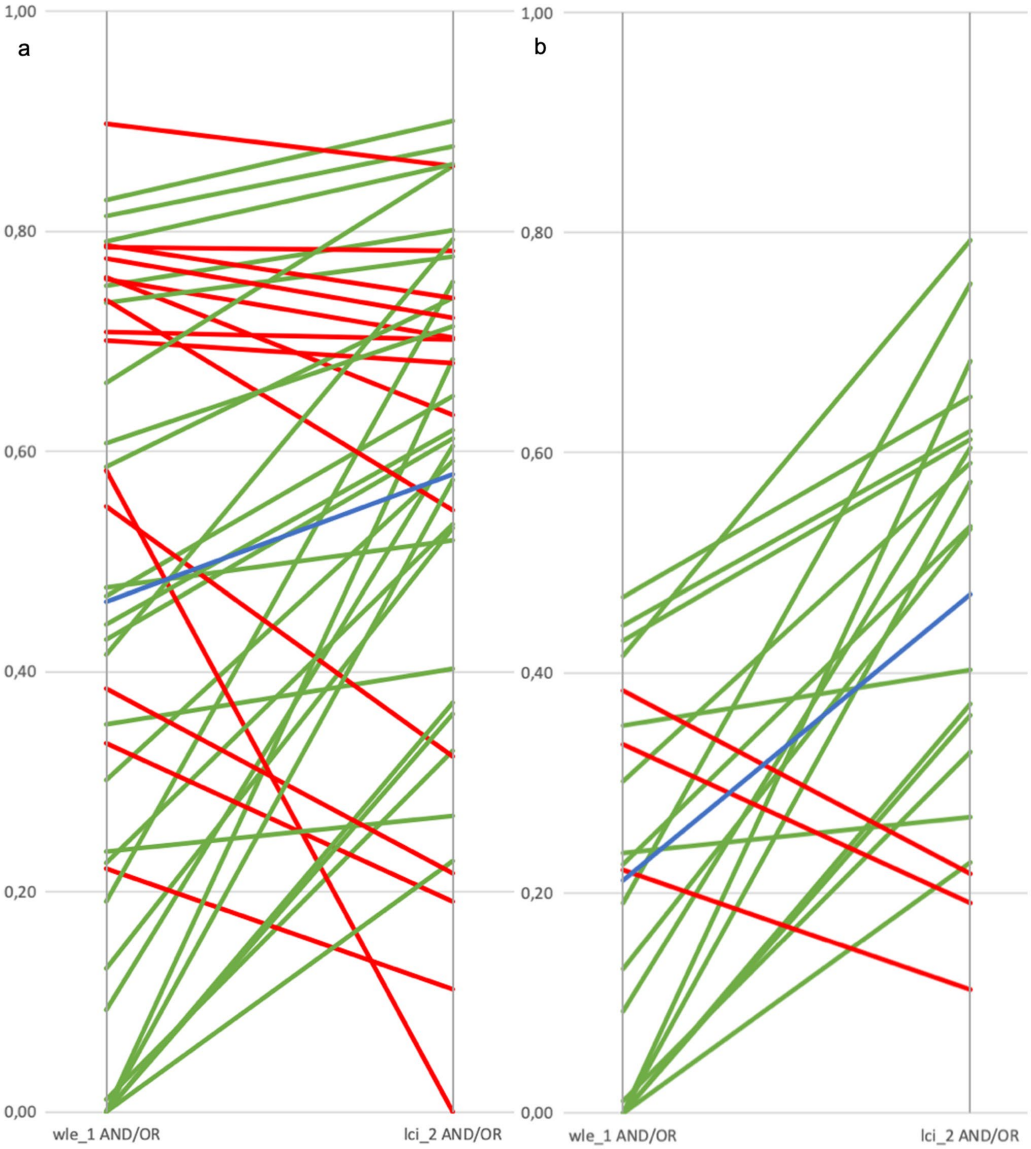
AND/OR ratios of phase 1 connected to the corresponding AND/OR ratio of phase 3 per case. Each line represents one individual case. Green lines: increase in AND/OR ratio, red line: decrease in AND/OR ratio, blue line: trend line, representing the mean effect between AND/OR ratios of both phases. **a** Showing all 40 cases. **b** Showing the 20 most challenging cases (i.e., with a WLE AND/OR ratio below the 50th percentile)

Median expert AND/OR ratios reflecting the level of agreement between the three experts increased significantly in phase 3 (WLE + LCI) when compared to phase 1 (WLE only) (0.58 vs. 0.46, respectively, *p* = 0.007; Table 1 and Fig. 3a).

**Table 1:** Results per assessment phase, for both expert endoscopists and non-expert endoscopists

	Phase 1 (WLE only)	Phase 2 (LCI only)	*p*-value	Phase 3 (LCI + WLE)	*p*-value
*Experts*					
Median AND/OR ratio*	0.47	0.65	0.009	0.63	0.007
Median AND/OR ratio sub analysis*	0.22	0.55	< 0.001	0.53	0.001
Median AND area*	30.000	35.053	0.851	39.807	0.055
Median OR area*	75.686	57.525	0.026	76.072	0.354
*Non-experts*					
Biopsy scores**	82.3%	83.2%	< 0.001	87.2%	< 0.001
Biopsy scores sub analysis**	70.4%	76.2%	< 0.001	83.4%	< 0.001
Ability to delineate the lesion*	5 (IQR 3–7)	6 (IQR 4–8)	< 0.001	7 (IQR 5–8)	< 0.001

Paired analyses using Wilcoxon-Signed Rank test (*) or McNemar test (**)

**Fig. 4:**
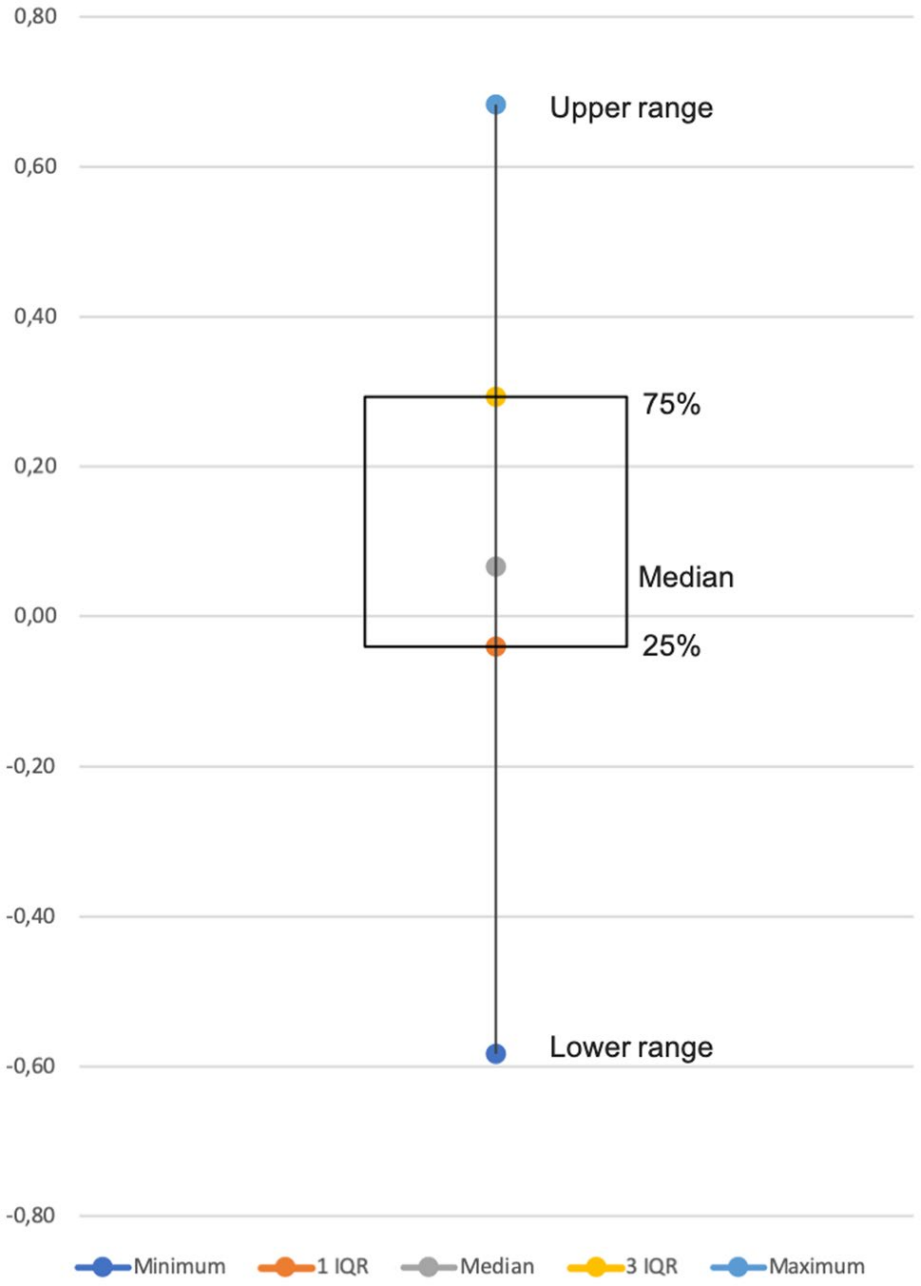
Boxplot graph of the scattering of differences in AND/OR ratios between WLE in phase 1 and LCI in phase 3 for all 40 cases

The differences between the individual AND/OR ratios of phase 1 and phase 3 were calculated (Fig. 4). This showed that in approximately 75% of the cases the AND/OR ratio increased when LCI is provided next to WLE images.

The increase in AND/OR ratio between phase 1 and phase 3 was even more apparent for the more challenging cases, i.e., the 50% of cases with a low baseline AND/OR score in phase 1 (i.e., low agreement between experts on WLE; Table 1). In 17 of the 20 cases, the AND/OR ratio improved when LCI was provided as an additional image (Fig. 3b).

The AND/OR ratio also increased significantly using only LCI (phase 2) when compared to using WLE only (phase 1) (median 0.65 vs. 0.47, respectively, *p* = 0.009).

### Non-expert performance

Seventy-three non-expert assessors started the first assessment phase. Of these 73 assessors, 62 assessors completed all three assessment phases of the study and were included for final analyses. The 62 assessors originated from four different countries (Japan 24, the Netherlands 10, Portugal 16, Sweden 12) and were divided in three different levels of endoscopic expertise: fellow (15), junior (21), and senior (26).

#### Biopsy mark placement

Correct biopsy placement increased significantly in phase 3 when compared to phase 1 (87.2% vs. 82.3%, respectively, *p* < 0.001; Table 1). Sub analysis showed an even more apparent increase (83.4% vs. 70.4%, *p* < 0.001; Table 1) for the more challenging cases (defined as cases with an AND/OR ratio of the experts WLE image assessment below the 50-percentile – see above).

Biopsy scores in phase 2 also increased significantly when compared to phase 1, yet the absolute increase was only minor (82.3% to 83.2%, *p* = 0.006; Table 1). There were no significant differences between the three groups of endoscopic expertise (data not shown).

#### Ability to delineate the lesion

The median VAS scores for ability to delineate the neoplastic lesion were significantly higher for phase 2 (LCI only) and phase 3 (WLE + LCI), compared to phase 1 (WLE only) (*p* < 0.001 for both phases, Table 1).

#### Preferred imaging modality

In phase 3, the non-expert endoscopists preferred the use of LCI over WLE in the majority of cases (72.4% vs. 5.2%, respectively, 22.4% no preference).

## Discussion

In this study, we demonstrate that LCI improves the visualization of early gastric cancer lesions over WLE for expert and non-expert endoscopists. The positive effect of LCI for the visualization of early gastric cancer has been described earlier [15, 16]; however, the benefit of this form of optical chromoscopy has not yet been evaluated for expert and non-expert endoscopists.

In the current study, the expert endoscopists reached a higher consensus in differentiating neoplastic tissue from the surrounding mucosa when using a combination of WLE and LCI. The positive additive effect of LCI was even more apparent for subtle neoplastic cases that were more difficult to delineate with WLE alone: in 85% of such cases, the AND/OR scores increased when experts had LCI at their disposal.

Experts delineated all neoplastic lesions in overview. It was not our aim to define the exact demarcation line of lesions, which is generally done with the use of magnified endoscopy and BLI prior to endoscopic resection. In our opinion, an increase in expert delineation overlap in overview indicates an improved identification of the most relevant part of the neoplastic lesion. We used the overlap area of our three expert delineations as a ground truth for non-expert assessments. It was our rationale that improved identification of early gastric cancer using LCI and would translate into better targeted biopsy acquisition by non-expert endoscopists. To evaluate this, non-expert endoscopists assessed the same endoscopic images as previously delineated by the experts and indicated their preferred biopsy location. Correct biopsy placement (i.e., placed within the overlap area of the three experts) increased significantly when both LCI and WLE were available. Again, this effect was even more prominent for the 50% most challenging cases on WLE. This indirectly indicates that with the addition of LCI to WLE, the non-expert endoscopists improved their discrimination performance between neoplasia and inflammation. The addition of LCI may therefore lead to an increase in primary detection of early gastric neoplasia, by providing a better distinction between neoplastic mucosa and surrounding inflammatory changes of the background atrophic gastritis. This translates into a higher target biopsy rate as shown in this study. The positive effect of LCI can be attributed to the fact that this imaging technique highlights the mucosal and vascular patterns slightly different compared to WLE. Finally, non-experts indicated that they strongly preferred the addition of LCI over WLE alone in their evaluation of cases.

The effect of LCI alone was also evaluated in this study. When comparing phase 1 with phase 2, the experts reached a higher agreement on the neoplastic lesions when using LCI alone, compared to WLE alone. A positive effect of LCI was also found for the non-experts, where the biopsy mark was placed within the experts’ overlap area more often than with WLE alone, although this effect was only minor. Based on the results of this study, the difference found between WLE alone and LCI alone was marginal with only little clinical relevance. In our opinion, LCI will primarily be used as an addition to inspection with white-light endoscopy. Therefore, this study relies on the evaluation of LCI as an addition to WLE for both expert endoscopists and non-expert endoscopists (i.e., phase 3 of our assessment).

This study has several strengths. Using the web-based module, we were able to quantify and compare the delineations of the experts for the different assessment phases and to create a ground truth for positioning of the biopsy mark by non-expert endoscopists. Due to the lack of availability of pixels precise correlation with the resection specimen, we reasoned that the overlapping area of the delineations of three expert endoscopists (the AND area) would include the area with the highest likelihood of neoplasia and therefore the preferred position for biopsy sampling. The delineation input of three Japanese expert endoscopists provided us with a reliable ground truth for non-expert assessment. Second, a large number of non-expert endoscopists from four different countries participated in this study, indicating robustness of our results.

Finally, the study design containing 3 separate assessment phases enabled us to directly compare the additive value of the imaging modalities.

This study also has several limitations. First, since only neoplastic cases were included in this study, we did not directly interrogate the additive value of LCI on the primary detection of early gastric cancer lesions. In future studies, we may include both neoplastic and non-dysplastic cases. Second, the retrospective design of this study might have led to selection bias. Images were only included when they were of sufficient quality and the WLE and LCI image corresponded to a high extent. Third, since the results of this study rely on ex vivo endoscopic images, the additional effect of LCI in daily practice has not been evaluated. Finally, preference for LCI of non-experts as assessed in the questionnaires may partly be caused by the fact that the non-experts were aware that they were participating in a study evaluating the effect of LCI. This may limit the external validity of this result. Future research might focus on the cost effectiveness of this new-generation endoscopy system.

In conclusion, this study shows that the addition of LCI next to WLE improves visualization and identification of early gastric cancer. Expert endoscopists reach a higher consensus on the discrimination between neoplasia and inflammation when using LCI. Non-expert endoscopists improve their targeted biopsy placement with the use of LCI. This effect is even more profound for lesions that are difficult to visualize with WLE. LCI therefore appears to be a useful tool in the identification of early gastric cancer.
